# Soil Surface-Trapping of Tomato Leaf-Miner Flies Emerging from Underground Pupae with a Simple Electrostatic Cover of Seedbeds in a Greenhouse

**DOI:** 10.3390/insects11120878

**Published:** 2020-12-11

**Authors:** Teruo Nonomura, Hideyoshi Toyoda

**Affiliations:** 1 Laboratory of Phytoprotection Science and Technology, Faculty of Agriculture and Agricultural Technology and Innovation Research Institute, Kindai University, Nara 631-8505, Japan; 2 Research Association of Electric Field Screen Supporters, Nara 631-8505, Japan; toyoda@nara.kindai.ac.jp

**Keywords:** static electric field, attractive force, greenhouse physical control, organic farming, pesticide-alternative pest control

## Abstract

**Simple Summary:**

Frequent infestation caused by tomato leaf miner flies (*Liriomyza sativae*) is a serious problem in the pesticide-independent cultivation of greenhouse tomatoes. This problem is caused by the persistent settlement of the flies in the greenhouse through larval movement between the phylloplane and rhizosphere soil of the host plants. The present work was conducted to develop a new physical control method to disrupt this developmental relationship. A simple electrostatic cover (EC) was constructed to trap adult flies emerging from underground pupae. The EC consisted of insulated iron rods linked to a voltage generator, which supplied a negative charge to the insulated iron rods, and non-insulated iron rods linked to a grounded line. The electric field formed in the space between the negatively charged and grounded iron rods generated an attractive force that could trap the target insects entering the electric field. A practical assay to demonstrate the functionality of the EC in a greenhouse revealed that the EC was able to capture all adult flies emerging from pupae. The simple structure of the EC makes it easy to fabricate for farmers who wish to integrate it into their pest management strategy. Thus, the present work provides an experimental basis for an electric field-based method for the control of tomato leaf miner flies.

**Abstract:**

In the present study, an electrostatic apparatus for trapping adult tomato leaf miner flies (*Liriomyza sativae*) emerging from underground pupae at the surface of a seedbed in an organic greenhouse was developed. The apparatus consisted of insulated iron rods arranged in parallel at set intervals and linked to a voltage generator, which supplied a negative charge to the rods, as well as non-insulated grounded iron rods with the same configuration. The two layers of insulated and non-insulated iron rods were arrayed in parallel to form a static electric field between the layers. The electric field created a strong attractive force capable of capturing flies that entered the field. In a greenhouse assay, the apparatus was placed horizontally above a seedbed in a greenhouse and surveyed for its ability to capture adult flies emerging from pupae that were introduced onto the seedbed beneath the apparatus. The results revealed that the apparatus effectively trapped all adult flies that emerged from the pupae and that it functioned stably while continuously operated during the entire period of the experiment. Thus, our novel apparatus is a promising tool for the physical control of adult tomato leaf miners in the insecticide-independent cultivation of greenhouse tomatoes.

## 1. Introduction

In our previous study on organic tomato cultivation in a plastic hoop greenhouse, the tomato plants frequently suffered attacks by the tomato leaf miner fly (*Liriomyza sativae*; Diptera: Agromyzidae) [1]. Leaf feeding by leaf miners resulted in white, winding mines, and heavily mined leaflets had large whitish blotches. Leaves injured by leaf miners dropped prematurely, and heavily infested plants occasionally lost most of their leaves. When mining occurred early in the fruiting period, defoliation reduced yields and fruit size and exposed the fruit to sunburn. The tomato leaf miner is normally a pest of late summer tomatoes and can infest plants in great numbers [2]. Additionally, in our district (Nara Prefecture, Japan), greenhouse tomatoes can be seriously damaged in the late summer season, and it is very important to control the miners using a pesticide-free method. Physical measures of pest control have been developed, based on electrostatics. In one basic method, a pest-free space is created in a glass greenhouse by installing an electric field screen (EFS) at all openings [3,4,5,6]. In addition, a simple and cheap version of an EFS (a bamboo blind-type EFS) was devised for plastic hoop greenhouses used by small farmers [7]. The EFS effectively prevented outside insects from getting into the greenhouse. However, in the case of tomato leaf miners, we were forced to consider additional countermeasures against pest attack.

The adult leaf miners deposit eggs in the leaf, and after hatching from the eggs, the larvae begin to eat tissues within the leaf and form extensive mines via tunneling. Eventually, the larvae crawl out of the leaf and fall to the ground. They then crawl into the ground where they pupate. Adult flies emerge from the underground pupae and oviposit eggs on host plants [8]. This life cycle can cause the persistent infestation of greenhouse tomatoes by this insect pest, thus necessitating additional countermeasures against adult flies emerging from the soil. To solve these problems, the soil bed was covered with a mulching sheet to keep adult tomato leaf miners from emerging. Unfortunately, mulch application is unsuitable for plant cultivation in the summer because of the undesirable enhancement of soil temperature.

Before initiating tomato cultivation in an organic farming system, we formerly rotated the application of insecticides, such as abamectin (a mixture of avermectin B1a and B1b) and cyromazine, the most commonly used insecticides for managing leaf miners on plant foliage [9], or drenched the soil beneath the plants with spinosad [10]. However, leaf miners rapidly evolved strains that were resistant to the previously effective insecticides. The failure of synthetic insecticides has also been reported in many other countries. In fact, many studies have reported the acquisition of resistance to major insecticides, such as abamectin [11], avermectin and cyromazine [9], fenpropathrin [12], chlorpyrifos [13], and spinosad [14], as well as a decrease in pesticide impact from parasitoids that control *Liriomyza* species [15]. This situation prompted us to change our pest control measures to insecticide-independent methods, with the goal of eco-friendly pest management, due to both the resistance problem and public concern about the use of agrochemicals.

The previous approaches of controlling phytophagous ladybird beetles using entomopathogenic bacteria that stably colonise tomato leaves [16,17,18] provided the impetus for initiating the biological control of tomato leaf miners. Kaspi and Parrella [19] reported that the most promising nonchemical approach for controlling *Liriomyza* leaf miners in greenhouses was the regular release of the parasitoid *Diglyphus isaea*, and that the augmentative release of the parasitoid together with sterile leaf miner males was more efficient than the use of either method alone. Using their method, we faced challenges in the use of parasitoid-based biocontrol of tomato leaf miners for several years. The approach was occasionally effective in keeping the population of leaf miners below the level of economic injury. However, the results were generally unstable year to year, probably because of the unsuccessful optimisation of parasitism for effective control, especially the failure to maintain *D. isaea* females at a high proportion caused by lower offspring fitness on tomato plants [20], which resulted in reduced host searching for oviposition and fewer feeding adults. These biological factors were closely related to each other, and it was therefore difficult to regulate them optimally. Eventually, a certain number of leaf miners survived and developed into a large population as new host plants became available. The lack of success with this approach provided a secondary motivation for developing a better control method for managing the pest. It seemed likely that a physical method would be suitable for this purpose. An electrostatic attractive force, which was first devised to precipitate the airborne conidia of phytopathogenic fungi [21,22], was considered a potential method for trapping target insects without any influence from biological or environmental factors.

In the present study, an electrostatic cover (EC) was constructed to place it over a seedbed in a greenhouse to trap adult tomato leaf miners emerging from underground pupae in the seedbed. The EC apparatus created an electrostatic field that generated an attractive force that captured flies entering the field. The configuration of the apparatus was simple, and it was easy to self-construct at low cost. In the present paper, we assessed the ability of the apparatus to capture adult tomato leaf miners and verified the feasibility of the present method as a physical pest control strategy.

## 2. Materials and Methods

### 2.1. Insects

Adults of the tomato leaf miner fly (*L. sativae* Blanchard) were used in the study. Pupae of the flies were purchased from Sumika Technoservice (Hyogo, Japan) and maintained in a growth chamber (25.0 ± 0.5 °C, 12 h photoperiod at 4000 lux) until adults emerged. The newly emerged adults were collected with an insect aspirator (Wildco, Yulee, FL, USA) and used for the following experiment.

### 2.2. Construction of the EC

**Figure 1:**
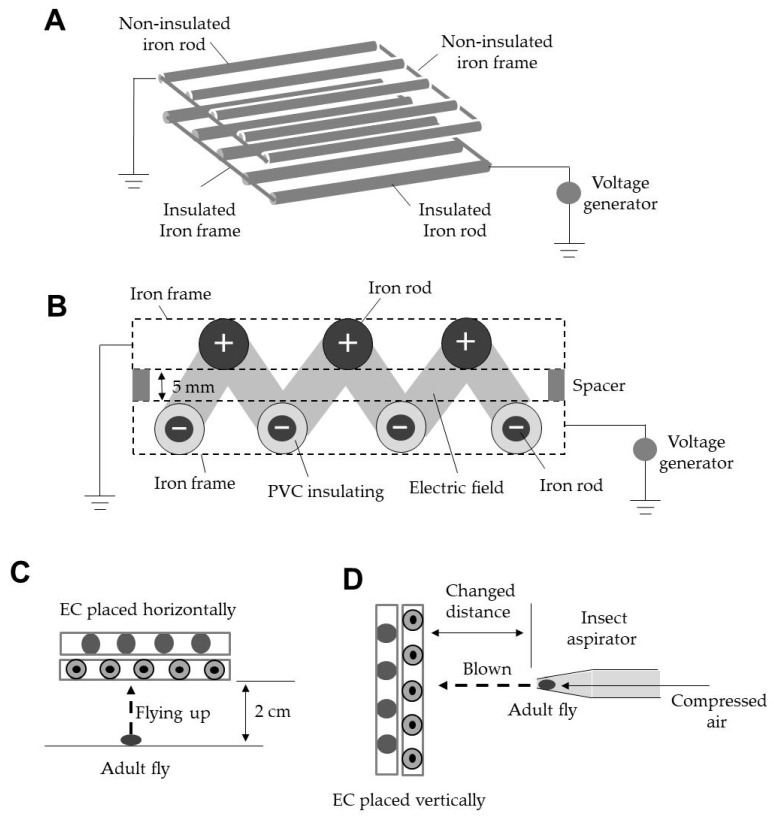
Diagram of the structure of an electrostatic cover (EC) for capturing adult tomato leaf miner flies emerging from underground pupae (**A**). An electrostatic field formed between negatively charged insulated iron rods and grounded non-insulated iron rods (**B**). One EC was placed horizontally to capture insects that fly upward (**C**), and other ECs were placed vertically to capture insects blown toward the EC (**D**).

Figure 1A shows the structure of the EC. The EC consisted of round iron rods and a direct current (DC) voltage generator (Max Electronics, Tokyo, Japan). The iron rods (length, 30 or 100 cm; diameter, 2 mm) were arrayed in parallel at fixed intervals and welded to a square iron frame (frame plate: thickness, 1 mm; height, 4 mm; length of square, 30 cm). The frame-fixed iron rods were coated with a polyvinyl chloride (PVC) resin (coating thickness, 1 mm; resistivity, 10^9^ Ω cm) for insulation (Sonoda Seisakusho, Osaka, Japan) and linked to a voltage generator to supply a negative charge to the insulated iron rods (IRs). Another set of frame-fixed iron rods (length, 30 cm; diameter, 4 mm), which were not insulated, was linked to a grounded line. The non-insulated grounded iron rods (NGRs) were used as an opposite pole. The IR and NGR layers were arranged such that the two layers were spaced 5 mm apart and offset from each other (Figure 1B). The negative charge of the IRs polarised the NGRs positively via electrostatic induction [23], and an electric field formed between the opposite charges of the IRs and NGRs. Insects entering this electric field were attracted to the negatively charged IRs as a result of the positive electrification of the insect body [24,25,26,27].

### 2.3. Insect Capture Assay

In the first experiment, the EC was placed horizontally at 2 cm above the floor and negatively charged with different voltages (between −1 and −6 kV). Adult flies were transferred onto the floor beneath the EC with an insect aspirator to determine whether the insects that flew upward were captured by the IRs (Figure 1C). Twenty flies were used for each voltage to determine the rate of insect capture at a given voltage. In addition, pupae of the tomato leaf miner were similarly placed beneath the EC, and the emergence of adult flies from the pupae and the subsequent capture of the flies by the EC were recorded on video.

In the second experiment, the EC was placed vertically and charged with negative voltages (−1 to −6 kV) to determine the voltage range within which all test insects were captured. Adult flies were blown into the space between the IRs and NGRs by passing compressed air (1.5 kg/cm^2^) through the tip of an insect aspirator (Figure 1D). The distance between the tip of the aspirator and the surface of the IRs was altered to create different wind speeds (1–3 m/s). Wind speed was measured at the surface of the IRs using a sensitive anemometer (Climomaster 6533; Kanomax, Tokyo, Japan). Twenty adults were used for each wind speed and voltage tested. The capture of adult flies was recorded on video.

In both experiments, a blower (max. wind speed at the IRs, 7 m/s) was directed at the captured insects for 10 min to confirm the successful capture of tomato leaf miners with the IRs. Both experiments were repeated five times, and the data are presented as the means with standard deviations. The statistical significance of data differences among treatments was analysed as described in the captions of Figures 3 and 4. All experiments were conducted in a room with the temperature controlled at 25 ± 1 °C.

### 2.4. Greenhouse Assay of the Practicality of the EC

**Figure 2:**
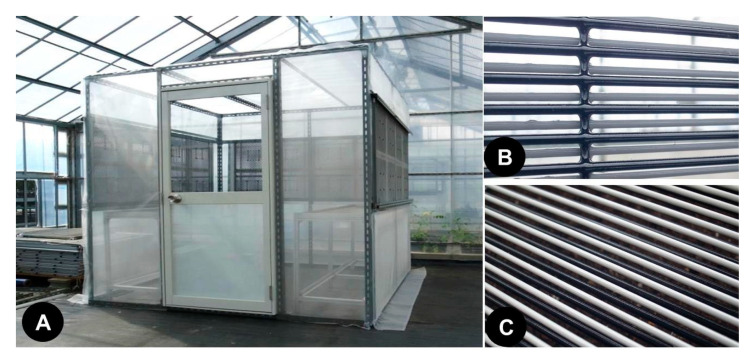
An experimental cabin furnished with electrostatic covers (ECs) in the windows of three walls (**A**). An EC installed in the wall of the cabin (**B**), and the EC placed over the seedbed in the cabin (**C**).

To assess the practicality of the EC, a rectangular cabin (4 × 4 × 2 m) was constructed and placed in a glass greenhouse. The cabin was covered with a semi-transparent vinyl sheet (Figure 2A), and ECs (1 × 1 m) were installed in three lateral windows of the cabin (three ECs per window) (Figure 2B). In the cabin, a seedbed (1 × 1 m) and 10 potted tomato plants (*Solanum lycopersicum* cv. Moneymaker; non-infested, 1-month-old seedlings) were placed on the floor. Twenty tomato leaf miner pupae were placed on a piece of white filter paper (30 × 30 cm) laid over the soil of the seedbed and then covered horizontally with an EC (1 × 1 m) (Figure 2C). The window and seedbed ECs were charged with voltages of −5.5 and −4 kV, respectively. This experiment was conducted to test four combinations of charged and uncharged ECs: (1) ECs at both locations charged, (2) window ECs charged and seedbed EC not charged and (3) vice versa, and (4) ECs at both locations not charged (Table 1). Three days later, the successful emergence of adult flies from test pupae was confirmed, and all test plants were transformed to a pest-free, closed greenhouse (maintained at 25 ± 2 °C) after all flies were eliminated from the plants. After 3 weeks of cultivation, the appearance of mines in the leaves was surveyed. At the end of the experiment, the number of the flies captured with the ECs was counted. For each treatment, the experiment was repeated three times. The present study was conducted from May to June 2020 (diurnal change in room temperature over the period of the experiment: 12–30 °C).

In the second experiment, both types of ECs in the cabin were charged continuously at the voltages mentioned above, and 20 pupae were placed on the paper beneath the seedbed EC every 4 days over the experimental period (1 month). At the end of the experiment, we surveyed the presence or absence of mines in the leaves of the test plants in the cabin.

## 3. Results and Discussion

The purpose of the present work was to devise a practical device for capturing adult tomato leaf miners emerging from the soil. For this purpose, we used an attractive force that could be generated in a static electric field. The major characteristic of the static electric field was the negative charge on the insulation of the conductor. The negative charge that accumulated on the surface of the conductor was not released (discharged) because of the surface insulation. However, the negative charge created a strong repulsive force for other negative charges (electrons) in the static electric field, thus pushing them toward the ground via the non-insulated grounded conductor. Through this mechanism, any conductor entering this field is deprived of its free (negatively charged) electrons and becomes electrified positively (positively charged). This phenomenon is called discharge-mediated positive electrification of a conductor [1]. Here, we were interested in how insects responded to the static electrtic field upon entering it. Recently, Takikawa et al. [28] reported that a static electric field can be used for insect control due to the water-mediated conductivity in the bodies of houseflies; thus, an insect that enters the static electric field is deprived of the free electrons in its body and becomes positively charged. This implies that discharge-mediated positive electrification can be induced in the insect, as the free electrons from the insect’s body move to the earth ground. Positively electrified insects are attracted to the insulated opposite conductor wire. This force is so strong that the captured insects cannot escape from the trap. This capture mechanism is applicable to almost all insects, as they tend to possess a conductive body. In fact, we demonstrated that, in a static electric field, different kinds of insects were similarly attracted to a negatively charged conductor as a result of positive electrification of the insect body, due to the loss of electrons from the body [24]. The insects tested were tomato leaf miner (*L. sativae*), greenhouse shore fly (*Scatella stagnalis*), bathroom fly (*Clogmia albipunctatus*), Asian tiger mosquito (*Aedes albopictus*) (Diptera), green peach aphid (*Myzus percicae*), whitefly (*Bemisia tabaci*), green rice leafhopper (*Nephotettix cincticeps*) (Hemiptera), rice weevil (*Sitophilus oryzae*), red flour beetle (*Tribolium castaneum*), adzuki bean weevil (*Callosobruchus chinensis*) (Coleoptera), oriental termite (*Coptotermes formosanas*) (Isoptera), book louse (*Liposcelis bostrychophilus*) (Psocoptera), German cockroach (*Blattella germanica*) (Blattodea), common clothes moth (*Tineola bisselliella*) (Lepidoptera) and western flower thrips (*Franklinella occidentalis*) (Thysanoptera).

Based on these results, this work focused on three points: (1) the ability to capture all soil-emerging flies, (2) a simple structure that can be fabricated easily at low cost by users, and (3) a sturdy structure that enables continuous operation during long periods of plant cultivation.

### 3.1. Functionality of the EC

The EC generated a gap-free electric field ‘zigzag’ between the IR and NGR arrays arranged in an offset manner (Figure 1B) such that the flies could not pass through the barrier of electric fields if the attractive force was strong enough to capture insects entering the field. The strength of the force depends on the voltage applied and the distance between opposite poles. In this case, the voltage was the causal factor because the distance between the IRs and NGRs was fixed.

**Figure 3:**
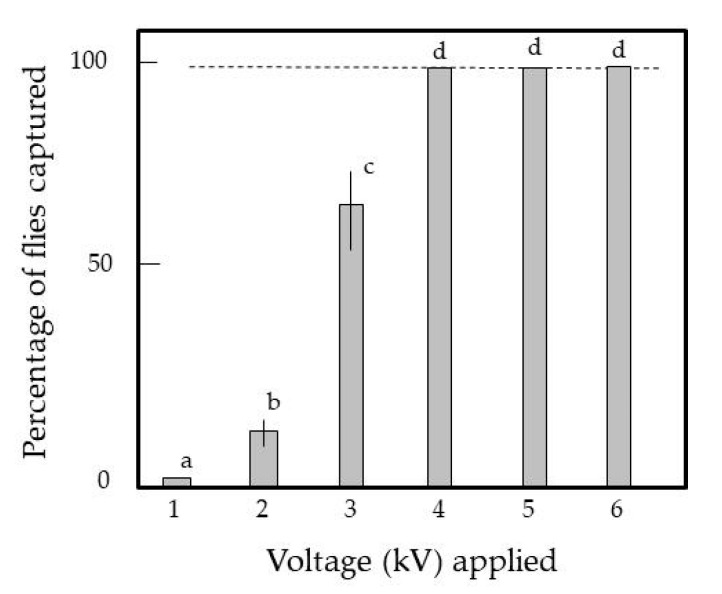
Capture of tomato leaf miners with the EC negatively charged with different voltages. Adult flies were placed on the floor beneath the EC. Twenty flies were used for each voltage applied, and the means and standard deviations were calculated from five repetitions of the experiments. The letters (a–d) in each column indicate significant differences (*p* < 0.05) according to Tukey’s method.

Figure 3 shows the percentages of adult tomato leaf miners captured by the EC at different voltages (−1 to −6 kV). The force became stronger with higher voltage applied to the IRs. At voltages greater than −4 kV, the force was strong enough that the IRs captured all tomato leaf miners; in fact, the force was strong enough to continue capturing tomato leaf miners despite a wind speed of 7 m/s. However, at lower voltages, the force was insufficient to capture tomato leaf miners permanently. The captured insects could flutter their legs or twist their bodies to escape and fly away from the IRs; otherwise, they were blown away from the IRs by the blower. Based on these observations, the IRs were charged with −4 kV in subsequent experiments to ensure successful capture. The mechanism of insect capture in the static electric field was described in previous work [24,25,26,27,29]. Video S1 shows the series of events from the emergence of adult flies from the pupae to the capture of the flies with the EC. For all pupae used, which were initially placed beneath the EC, the adult flies that emerged walked around for several minutes and then flew upward. These flies were successfully captured by the IRs of the EC under the voltage conditions mentioned above. These results suggest that the present method can be applied to capture adult tomato leaf miner flies that were initially enclosed beneath the ground [8] before appearing on the surface of the seedbed.

### 3.2. Evaluation of the Feasibility of the EC under Greenhouse Conditions

Before the present study, the populations of adult tomato leaf miners in some tomato greenhouses had been extremely large and had repeatedly attacked the tomato plants. Given this situation, there is a significant risk that adult offspring could move to neighbouring greenhouses in search of new host plants. In fact, in the glass greenhouse used for the present study, some tomato plants were attacked and exhibited typical mines in some leaves. The glass greenhouse was ventilated continuously by several ventilating and circulating fans; therefore, the cabins constructed in this glasshouse were also exposed to a continuous airflow of 1–3 m/s. In the present study, the ECs were installed in the lateral windows of the cabin to prevent adult leaf miners from invading the cabin through the windows. Under the present greenhouse condition, each EC was required to capture the adult flies carried by wind with a maximum velocity of 3 m/s. To determine the optimal voltage, adult flies were blown toward the EC when it was negatively charged at different voltages.

**Figure 4:**
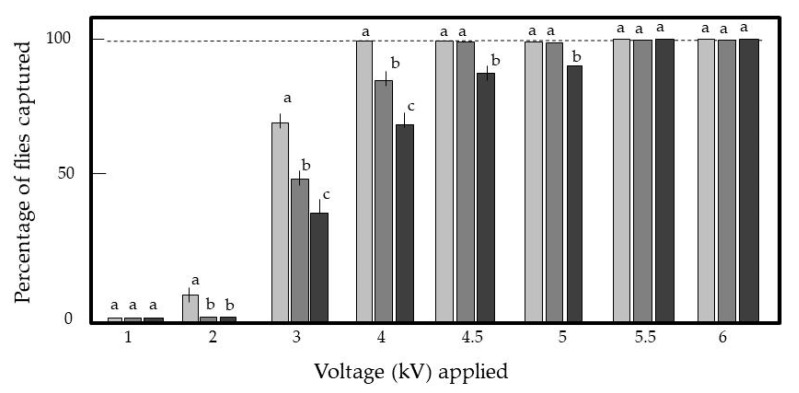
Capture of tomato leaf miner flies with the insulated iron rods negatively charged with different voltages. Adult flies were blown inside the space of the negatively charged insulated iron rod and the grounded iron rod at different wind speeds. The triplet columns represent 1, 2, and 3 m/s, from left to right, respectively. Twenty flies were used for each voltage and each wind speed, and the means and standard deviations were calculated from five repetitions of the experiments. The letters (a–c) in each triplet column indicate significant differences (*p* < 0.05) according to Tukey’s method.

Figure 4 shows the percentages of adult tomato leaf miners captured by the EC at different voltages (−1 to −6 kV) at each wind speed. Stronger forces from the IRs were required to capture tomato leaf miners carried at higher wind speeds, and the force increased with the increasing voltage applied to the IRs. At voltages exceeding −5.5 kV, the force was strong enough that the IRs captured all tomato leaf miners irrespective of the wind speed. Furthermore, the force was strong enough to keep capturing tomato leaf miners at a wind speed of 7 m/s. Video S2 shows the successful capture of adult leaf miners blown at a wind speed of 3 m/s. However, at voltages lower than −5.5 kV, the force was insufficient to permanently capture the flies. From these results, some important conclusions were derived: (1) at a voltage of −5.5 kV, the IRs of the EC exerted a force sufficient to capture tomato leaf miners blown inside the electric field at 3 m/s (the maximum wind speed inside the glass greenhouse), and (2) the captured tomato leaf miners could not escape the IRs even when they were blown at 7 m/s.

The aim of the present work was to propose a new physical control method for trapping tomato leaf miners emerging from underground pupae at the soil surface. Thus, the focus of the present greenhouse assay was to demonstrate that tomato plants remain uninfested if the seedbed EC is able to trap emerging adult flies at the soil surface. To evaluate the feasibility of the method, it was essential to exclude the participation of tomato leaf miners from outside. The cabin with ECs installed was used to exclude outside flies from the internal space of the cabin. The cabin constructed in the present study had ECs installed in the windows in three lateral walls to maintain good airflow. In fact, the spacing between the IRs and NGRs was wide enough to ensure good air permeation as well as strong insect-capturing ability. In this experimental setup, all adult flies inside the cabin could be regarded as flies that had emerged from the pupae placed on the paper.

Table 1 lists the results of the four experiments carried out to evaluate the feasibility of the present apparatus. First, in all experiments, we confirmed that adult flies emerged from all pupae by examining the castoff remnants within 3 days after the start of the experiments, and that the charged seedbed EC successfully trapped all emerged flies (Experiments 1 and 2 in Table 1). Moreover, the experimental results indicated that a few flies visited the cabin; the charged window ECs occasionally trapped adult flies that came from outside the greenhouse (Experiment 1) or a few plants were attacked by the flies and exhibited typical mines in their leaves (Experiment 2). Experiment 3 showed that the adult flies emerging from the pupae caused severe damage to test plants if they were not trapped at the soil surface. Judging from the significant difference in the total number of infested leaves of the test plants in Experiments 3 and 4, the plants in Experiment 4 suffered much heavier damage due to the double attack by both external and internal (pupae-derived) leaf miners. In our preliminary assay, *L. sativae* females were shown to produce more than 150 larvae at 25 °C on tomato plants during an average lifespan (14 days), and to exhibit peak fecundity at age 4–5 days. In the present study, younger adults (age 1–3 days) were used for larval production. However, their ability to oviposit was sufficient to obtain reliable data in the present comparative assay. In summary, the electrostatic apparatus devised in the present study was an effective tool for controlling the adult tomato leaf miners that emerged from underground pupae, as well as those that were carried through the window.

The functional durability of the ECs was confirmed by their continuous operation for one month. During this period, the seedbed EC trapped all adult flies emerging from pupae, which were introduced repeatedly at set time intervals (days), and the window ECs captured adult flies visiting from outside and prevented their entry. Due to the stable function of the ECs, the tomato plants in the cabin remained uninfested throughout the entire period of the experiment.

### 3.3. Simplicity and Durability of the EC

In our project to support small farmers in the suburban area of a large city, technical guidance is one of the main activities. In fact, the present work was planned in response to the actual requirements of farmers. Our fundamental response was to introduce new technical methods that can be easily taken up by farmers. Based on this concept, the present apparatus was proposed for practical implementation. The framework of the apparatus was simple and easy to fabricate with ubiquitous metal materials. The voltage generator was the only electrical instrument that needed to be purchased. It can be operated with a 12 V storage battery. The voltage generator can also be used to boost the initial voltage (12 V) to the designated voltage (1–30 kV) using a transformer (coil) and Cockcroft circuit integrated into an electric circuit in the voltage generator [30]. Two configurations for the voltage generator are commercially available: fixed voltage and adjustable voltage. During our investigations, the adjustable voltage model was used to examine the relationship between the voltages applied and the occurrence of electrostatic phenomena. Ultimately, a fixed voltage model was used in later experiments due to the lower cost of this type of generator.

In the laboratory-scale experiments, the insulated conductor was made by passing a metal wire through a soft PVC tube [29]. The insulated conductor is easy to construct, and there are no problems with its functionality. However, in the outdoor experiments with longer exposure periods, the installed electric field screens became susceptible to serious deterioration, such as discolouration, deformation, and cracking due to changes in temperature, humidity, and ultraviolet irradiation levels. These issues limit the practical implementation of the electrostatic apparatus [31]. To solve this problem, the conductors were coated with PVC resin to prolong apparatus operation in outdoor environments with minimal deterioration. In fact, this coating made it possible to operate the apparatus normally and continuously throughout the entire period of the experiment (1 month). Spraying iron rods with proper insulating paint may be another simple method for insulating the charged body of the apparatus.

## 4. Conclusions

The tomato leaf miner is a serious nuisance in pesticide-free greenhouse tomato cultivation. In contrast to other greenhouse pests that can be controlled by preventing their entry into a greenhouse, tomato leaf miners require another type of pest control management because of their persistent settlement in the greenhouse through larval movement between the phylloplane (for adults, eggs, and larvae) and rhizosphere soil (for larvae and pupae) of host plants. The EC was devised for this purpose. The present device was an apparatus designed to create a static electric field that would generate an attractive force for capturing target insects that entered the electric field. The EC was placed on the seedbed to trap adult tomato leaf miners emerging from underground pupae. The structure was simple and sturdy, so it could be easily fabricated by farmers and operated durably for long periods of time in a greenhouse. The ability to capture adult tomato leaf miners was substantial; therefore, this apparatus presents a promising alternative to pesticide use for controlling the tomato leaf miner.

## Figures and Tables

**Table 1:** Trapping of adult tomato leaf miners emerging from pupae by electrostatic cover (ECs) set in a cabin and survey of mine formation on leaves of tomato plants placed in a cabin ^a^.

Experiment	Charging of W-ECs at −5.5 kV	Charging of S-EC at −4 kV	Number of Flies		Number of Plants Infested ^b^	Number of Total Leaves Infested ^b^
			Captured with W-ECs	Captured with S-EC		
1	yes	yes	1.6 ± 0.2	20	0	0
2	no	yes	0	20	1.5 ± 0.3 v	4.0 ± 0.2 v
3	yes	no	1.4 ± 0.3	0	8.8 ± 0.4 w	66.0 ± 0.5 w
4	no	no	0	0	9.2 ± 0.2 w	96.0 ± 0.6 x

^a^ A seedbed was covered with the EC (S-EC) and placed in a cabin with lateral windows furnished with ECs (W-ECs). Ten potted tomato seedlings were placed in the cabin. Twenty pupae were placed on a paper laid on the seedbed and beneath the S-EC. The means and standard deviations were calculated from three repetitions of the experiments. The letters (v–x) in each vertical column indicate significant differences (*p* < 0.05) according to Tukey’s method. ^b^ Tomato plants were placed in a cabin while adult flies emerged from all pupae (for 3 days) and were then transferred to a pest-free greenhouse. Three weeks later, the plants were surveyed for the appearance of mines in their leaves.

## Supplementary Materials

The following are available online at https://www.mdpi.com/2075-4450/11/12/878/s1, Video S1: Emergence of an adult tomato leaf miner from a pupa and capturing of the fly with an insulated iron rod of the horizontally placed S-EC (–4 kV charge). Video S2: Capture of adult tomato leaf miners with the W-EC placed vertically (–5.5 kV charge). The flies were blown toward the EC at 3 m/s.
